# Association of Household Type and Fast-Food Consumption in Korean Adolescents

**DOI:** 10.3390/nu14153024

**Published:** 2022-07-23

**Authors:** Hwa Sook Kwon, Soo Hyun Kang, Yu Shin Park, Jung Gu Kang, Eun Cheol Park

**Affiliations:** 1 Department of Administration, CHA Ilsan Medical Center, Seoul 10414, Korea; flower21_@naver.com; 2 Department of Public Health, Graduate School, Yonsei University, Seoul 03722, Korea; kshyun@yuhs.ac (S.H.K.); dbtls0459@yuhs.ac (Y.S.P.); 3 Institute of Health Services Research, Yonsei University, Seoul 03722, Korea; 4 CHA Ilsan Medical Center, Seoul 10414, Korea; kangski1958@gmail.com; 5 Department of Preventive Medicine, College of Medicine, Yonsei University, Seoul 03722, Korea

**Keywords:** household, fast food, adolescents, dietary behavior, family

## Abstract

Background: Due to changing household types and weakening of family functions, children have fewer opportunities to develop healthy lifestyle patterns from contact with family members compared to the past. In this paper, we evaluate the association between household type and adolescents’ fast-food consumption, focusing on whether they were living with their parents or not, and determine their reasons for not living with their parents. Methods: This cross-sectional study analyzed data from the Korea Youth Risk Behavior web-based survey between 2017 and 2020. The subjects were students in grades 7–12. The outcome variable was a frequency of fast-food intake of ≥5 times per week. The main independent variable was the type of household: (1) living with both parents; (2) living with a single parent (one of father, mother, stepfather, stepmother); (3) not living together, but having parents; and (4) having no parents. Results: Participants without parents were more likely to eat fast food frequently than those living with both parents. Among boys, not having parents and living in a dorm or boarding house or living with other family members or relatives were significantly associated with frequent fast-food intake; among girls, not having parents and living in a dorm or boarding house were significantly associated with frequent fast-food intake. Conclusion: Adolescents having no parents have a higher risk of frequent fast-food intake than those living with both parents. Further studies are needed to address household types in greater detail.

## 1. Introduction

Consumption of fast food, such as instant noodles, hamburgers, pizza, fried foods, and sugar-sweetened beverages, is reported to be higher in adolescents than in individuals of other age groups [1]. Fast food is directly related to total energy intake and is reported as a factor that degrades the quality of a diet [2,3,4]. As fast foods are commonly consumed with soda, the risk of being overweight, risk of obesity, and the risk of consuming an unbalanced diet may increase due to soda consumption [5,6]. Korean adolescents often eat out because they spend a lot of time outside their homes for after-school academies [7]. As the food industry grows and food choices available at convenience stores have become extensive, children are being increasingly exposed to substantial amounts of fast food. Similar to adolescents in other countries, the increasing dietary consumption of fast food (which is convenient, low cost, and easily available) among adolescents in Korea has raised concerns regarding nutritional imbalance and hindered growth and development [8]. The “Let’s Move” program was launched in the US to fight childhood obesity and improve school lunches in February 2010, and stated that “as fast food became our everyday meal, children have been suffering from obesity and diabetes, which leads to several adult diseases” [1].

Adolescents’ dietary behaviors are established through a complex interaction process which involves both internal factors (such as food preference, availability, and appearance) and external factors (such as the influence of parents, peers, and income level) [9]. If their dietary behavior is inappropriate, the growth, development, and nutritional health of adolescents, as well as their life-long health, may be permanently impaired [10,11]. Inappropriate dietary patterns formed in childhood tend to persist into adulthood, which has a considerable effect on adults’ health and well-being [12,13,14]. Therefore, those family members who share the largest proportion of childhood have an immense influence on establishing adolescents’ dietary patterns.

Changes in household types are a global trend; the number of households composed of couples and unmarried children in Korea has continually declined from 58.0% in 1990 to 29.1% in 2020, whereas the number of households comprising single parents has increased from 8.7% in 1990 to 9.7% in 2020 [15,16]. In recent years, the proportion of households composed of various configurations, such as grandparents-raising-grandchildren households (0.6%), no-children households (16.9%), and multicultural households (1.8%), has been increasing [16]. Accordingly, the functions of the family have been downscaled. Due to weakening of family functions and kinship, opportunities for children’s lifestyle patterns to develop through observation, imitation, and learning from contact with family members have been reduced [17].

Therefore, this study aimed to examine the association between household type and adolescent fast-food consumption, focusing on whether they were living with their parents as well as their reasons for not living with parents.

## 2. Materials and Methods

### 2.1. Data and Study Population

Data for this cross-sectional study were obtained from the Korea Youth Risk Behavior Web-Based Survey (KYRBWS), which was conducted annually from 2017 to 2020 by the Korea Centers for Disease Control and Prevention (KCDC). The requirement for ethics approval from the KYRBWS was waived by the KCDC’s institutional review board in accordance with the Bioethics and Safety Act of 2015 [18,19]. All data used in this study are publicly available on the KYRBWS website [20]. The KYRBWS complied with the Declaration of Helsinki [21], and all individuals who participated in the KYRBWS provided informed consent.

The KYRBWS used an anonymous and self-administered structured questionnaire with a complex research design that included multistage sampling, stratification, and clustering. The KYRBWS used an online survey system that did not allow respondents to proceed to the next section of the questionnaire unless all questions in the current section were answered. Responses that had logical errors or were outliers (e.g., the respondent incorrectly stated that he or she was younger than 12 years, and thus could not have been included in the KYRBWS because of its grade-level criteria) were processed as missing values. The questionnaire comprised approximately 120 items across 15 categories, including demographic characteristics and health-associated behaviors. The validity and reliability of this survey have been confirmed previously [22,23]. Students in grades 7 to 12 were the target population.

### 2.2. Variables

The outcome variable was the frequency of fast-food intake per week. All participants answered the question, “How often have you eaten fast food in the last seven days?” A frequency of never to four times a week was classified as “not eating often,” whereas more than five to six times a week was classified as “eating frequently”.

The main independent variable was household type. The type of cohabitation with parents was first evaluated by choosing all the current family members, then by additionally asking whether they lived with their father (including stepfather) and whether their father and mother (including stepmother) lived together. Family types were classified into four types according to whether they lived with their parents or not: (1) living with both parents; (2) living with a single parent (one of father, mother, stepfather, stepmother); (3) not living together but having parents; and (4) having no parents. For the secondary analyses, the two groups not living with parents were divided into three subgroups based on whom they lived with and where they lived: (1) living with other family members or relatives; (2) living in a dorm or boarding house; and (3) living in an orphanage.

The covariates included the survey year (2017–2020), school grade (7–12), self-reported economic status (low, middle, or high), residential areas (capital area, urban, or rural), smoking status (yes or no), alcohol use (yes or no), body mass index (under-weight, normal, or obese), physical activity (yes or no), and depressive symptoms (yes or no). Physical activity was defined using the following question: in the last seven days, on how many days has your heart rate increased more than usual or have you been out of breath due to physical activity (regardless of type) for a total of 60 min or more per day? Depressive symptoms were assessed using the following question: during the last 12 months, have you felt sadness or despair that interrupted your daily life for at least two weeks?

### 2.3. Statistical Analysis

All analyses were conducted separately by sex to account for sex-specific differences in dietary behavior. Differences in the frequency and proportion of the categorical variables were evaluated using the χ2 test. Multiple logistic regression analysis was performed to examine the association between household type and adolescents’ fast-food consumption patterns, with adjustment for covariates to calculate the adjusted odds ratios (OR) and 95% confidential intervals (CI). All statistical analyses were performed using SAS software version 9.4 (SAS Institute), and used weighted logistic regression to account for the population’s representative, clustered, and stratified sampling design. The results were considered statistically significant if the *p*-value was <0.05.

## 3. Results

A total of 245,839 students participated in the KYRBWS from 2017 to 2020. After excluding participants with missing values (*n* = 59,562), the final study population was 186,277 students (56,767 students in 2017, 55,124 in 2018, 34,990 in 2019, and 39,396 in 2020).

This study included 186,227 adolescents (89,280 boys and 96,997 girls) (Table 1). Among them, 3992 boys (4.5%) and 3505 girls (3.4%) reported consuming fast food five times or more in the last seven days. Among boys, 74,992 (85.6%) lived with both parents, 12,016 (13.7%) lived with a single parent, 1477 (1.7%) had parents but were not cohabitating, and 795 (0.9%) did not have parents. Among girls, 81,917 (85.3%) lived with both parents, 13,004 (13.5%) lived with a single parent, 1554 (1.6%) had parents but were not cohabitating, and 522 (0.5%) did not have parents. Among boys, the frequency of fast-food consumption was higher among those who did not have parents (9.1%) than among those living with both parents (4.3%), living with a single parent (5.0%), and living apart from their parents (5.1%). Among girls, the rate of fast-food consumption was higher among those who did not have parents (7.3%) than among those living with both parents (3.5%), living with a single parent (4.1%), and living apart from their parents (4.5%). Generally, both boys and girls had significantly different characteristics. However, there were no statistically significant regional differences in either boys or girls, or in physical activity among boys.

Participants who had no parents were more likely to eat fast food frequently than those who were living with both parents (boys: OR, 2.00, 95% CI, 1.52–2.62; girls: OR, 1.58, 95% CI, 1.09–2.30) (Table 2). The probability of frequent fast-food intake was increased among those who were living with a single parent (boys: OR, 1.08, 95% CI, 0.98–1.19; girls: OR, 1.03, 95% CI, 0.93–1.15) or living apart from their parents (boys: OR, 1.04, 95% CI, 0.80–1.35; girls: OR, 1.18, 95% CI, 0.89–1.62), although it was not statistically significant.

In subgroup analyses stratified by covariates, boys without parents had a strong association with frequent fast-food intake when they lived in a low- (OR, 2.60, 95% CI, 1.67–4.07) or high -income household (OR, 1.81, 95% CI, 1.16–2.83) in 2017 (OR, 2.35, 95% CI, 1.50–3.70) and in 2018 (OR, 2.26, 95% CI, 1.47–3.47) (Table 3). Boys living with a single parent in a high-income household had an increased risk of frequent fast-food intake (OR, 1.22, 95% CI, 1.02–1.46). Among girls without parents, there was a strong association with frequent fast-food intake when they had a low (OR, 2.13, 95% CI, 1.24–3.64) or high household income (OR, 2.30, 95% CI, 1.11–4.80).

Table 4 shows the secondary analyses regarding whom the participants were living with and where they were living for participants not living with their parents. Boys who did not have parents and were living in a dorm or boarding house (OR, 4.58, 95% CI, 2.27–9.23) or living with other family members or relatives (OR, 1.85, 95% CI, 1.36–2.53) demonstrated a significant association with frequent fast-food intake compared to those living with both parents. For girls, those who did not have parents and were living in a dorm or boarding house (OR, 4.40, 95% CI, 1.75–11.08) had a significant association with frequent fast-food intake compared to those living with both parents. Generally, participants living in other types of households had an increased risk of frequent fast-food intake compared to those living with both parents, although the statistical significance was marginal. Boys who lived apart from their parents with other family members or relatives and girls who lived apart from their parents in an orphanage had a decreased risk of frequent fast-food intake, although the statistical significance was again marginal.

**Figure 1:**
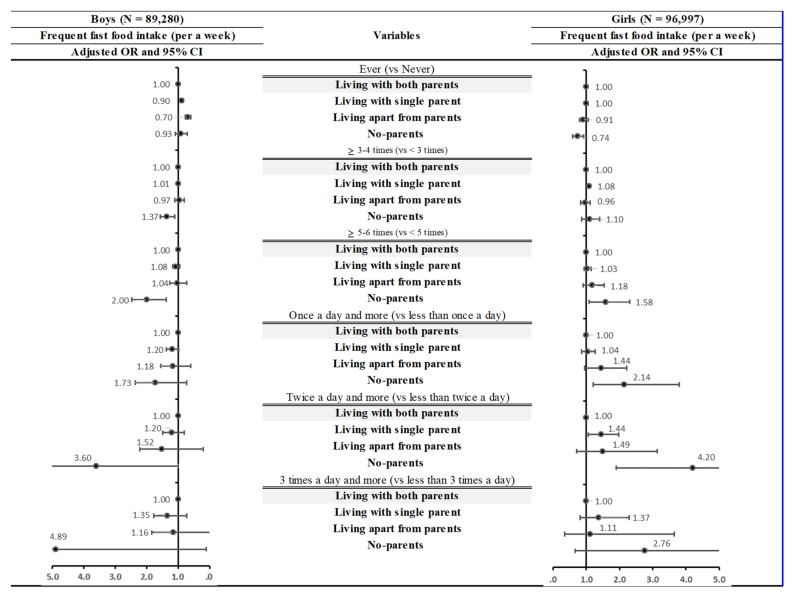
Results of sensitivity analysis by defining fast food intake. All covariates were adjusted. OR: Odds ratio; 95% CI: 95% Confidential Intervals.

Subsequently, sensitivity analyses were conducted to confirm the robustness of the results regardless of the changing definition of frequent fast-food intake (Figure 1). In analyses with various cut-offs for defining the frequency, participants who lived with a single parent, apart from their parents, or who had no parents had an increased risk of frequent fast-food intake, except in the analysis of ever versus never fast-food intake. Although the statistical significance was marginal in a few analyses, participants who did not have parents had the highest risk of frequent fast-food intake in most analyses.

## 4. Discussion

After adjusting for demographic, socio-economic, and health behavior factors, adolescents who did not have parents had a higher risk of frequent fast-food intake than those who lived with both parents. Among adolescents without parents, boys living in a dorm or boarding house or living with other family members or relatives as well as girls living in a dorm or boarding house had a notable association with frequent fast-food intake. Furthermore, regardless of the various definitions of frequency of fast-food intake, adolescents without parents were the most likely to consume fast-food.

In line with our study findings, the findings of previous studies have shown that parents play an instrumental role in establishing adolescents’ dietary behavior. Family, especially parents, are the first subjects whose behavior is mirrored after birth by their children. Moreover, they are the individuals who the children imitate before they are old enough to attend a day care center. Children learn mainly through observation and imitating what they observe. Therefore, parents’ words and deeds have a deep and subtle impact on children, and have a long-term impact on their adult life [17]. In a functioning family [24] or when children are involved in family meal preparation [25], children’s dietary behaviors are more likely to be healthier. Both parents and grandparents play an influential role in the development of healthy dietary behavior.

In an Australian study, grandparents helping with childcare were found to play a role in preventing obesity-related behavior in young children by restricting access to certain foods, allowing grandchildren a high degree of input and control when planning mealtimes and food choices, and providing more encouragement of a balanced dietary intake compared to parents [26]. Grandparents can play the role of surrogate parents on behalf of children’s real parents due to changes in household types, such as double income families, single-parent families, grandparents-raising-grandchildren families, and sibling-breadwinner families. Because of data limitations, we could not identify more detailed household types. However, we assume that certain factors, such as help from other family members or surrogate guardians on behalf of the parents, might influence the results in the analysis of household types. Further studies are needed to investigate household types in greater detail.

A previous study has reported that single-parent families are associated with a higher probability of an unbalanced diet than both-parent families. Due to the absence of dietary management by parents, children’s meals are more likely to be replaced by fast food. In particular, in low-income households, irregular food intake such as binge eating and excessive intake of low-nutritional and high-calorie foods such as fast food and snacks were shown to be prevalent due to neglect by parents or guardians [27]. The higher risk of frequent fast-food intake among adolescents without parents found in our study might be explained by the absence of dietary management and neglect by parents or guardians.

Considering that adolescents in low-income households, especially adolescents without parents, are more likely to eat fast food, we speculated that adolescents in orphanages would be more likely to eat fast food than those living with family or living in a dorm due to their limited financial capacity to choose healthy foods. However, adolescents without parents and living in a dorm or boarding house were strongly associated with frequent fast-food intake. Care providers’ supervision of eating behavior might be an explanation. In residential care facilities for children, regulations and supervision by the staff have an impact on the children’s dietary patterns [28,29]. On the other hand, living in a dorm surrounded by peers might be associated with an increase in fast-food intake due to inexpensive price, better accessibility, and less supervision by guardians [30].

In Korea, a “children’s meal card” (called the “Kkum-namu card”) has been provided to children from low- income, single-parent, or near-poor families beginning in 2009, allowing them to have meals outside of school whenever have not eaten school meals [31]. It has been pointed out that the minimum amount of one meal with the “Children’s meal card” is USD 3; this amount was very low for an appropriate meal, even in 2017. It was not enough to have a meal at restaurants, and was only enough for instant noodle cups or snacks in convenient stores. Hence, the minimum amount was increased to approximately 4 dollars in 2018 and then to 5, which was graded by age, in 2020. However, boys who were living with a single parent and girls who were living apart from parents in 2020 continued to have a higher risk of frequent fast-food intake in our study. Furthermore, to the best of our knowledge there is no evidence to show that the revised minimum amount of the “children’s meal card” is sufficient to reduce these children’s food insecurity. Therefore, further studies that examine the effect of the “children’ meal card” on children’s healthy and balanced diets are needed. Furthermore, more practical revisions or initiatives should be implemented to guarantee a well-balanced diet that reflects real-world costs.

This study has limitations. We could not identify the direction of the associations or establish causality, although appropriate methods were used to mitigate these limitations. Furthermore, the KYRBWS data were collected anonymously online and self-reported, which may have resulted in misclassification or biased results. Because the KYRBWS assessed cohabitation only with parents, not with grandparents or siblings, we could not evaluate whether the type of main guardian (i.e., grandparents, brother or sister, or other) was associated with fast-food intake among adolescents not living with their parents. In addition, certain associations had wide 95% CI or marginal statistical significance due to the small sample size, especially among those not living with parents.

Despite these limitations, this study identified the effect of both the household type and of the individuals adolescents were living with and where they were living on the frequency of fast-food intake. Furthermore, the multiyear national survey data (2017–2020) were obtained from approximately 186,000 adolescents based on random cluster sampling, ensuring that the results were sufficiently representative of Korean adolescents [32].

In this cross-sectional study, adolescents who did not have parents had a higher risk of frequent fast-food intake than those who lived with both parents. Among adolescents without parents, boys living in a dorm or boarding house or living with other family members or relatives and girls living in a dorm or boarding house had a notable association with frequent fast-food intake. In analysis with various definitions of frequency of fast-food intake, adolescents without parents were the most likely to consume fast food. These findings suggest that the type of household and cohabitation are associated with the frequency of fast food intake among adolescents. This research can provide evidence related to healthy dietary patterns among Korean adolescents. Consideration should be paid to the person(s) with whom they were living with and where they were living when conducting future research, as well as when revising public policies regarding well-balanced diets for children.

## Figures and Tables

**Table 1:** General characteristics of the study population.

Variables		Frequent Fast-Food Intake													
		Boys							Girls						
		Total		Yes		No		*p*-Value	Total		Yes		No		*p*-Value
		N	%	N	%	N	%		N	%	N	%	N	%	
	Total 186,277	89,280	100.0	3992	4.5	85,288	95.5		96,997	100.0	3505	3.6	93,492	96.4	
Type of Household															
	Living with both parents	74,992	85.6	3243	4.3	71,749	95.7	<0.0001	81,917	85.3	2865	3.5	79,052	96.5	<0.0001
	Living with single parent	12,016	13.7	602	5.0	11,414	95.0		13,004	13.5	532	4.1	12,472	95.9	
	Living apart from parents	1477	1.7	75	5.1	1402	94.9		1554	1.6	70	4.5	1484	95.5	
	No parents	795	0.9	72	9.1	723	90.9		522	0.5	38	7.3	484	92.7	
Grade															
	Middle school 1st	15,932	18.2	605	3.8	15,327	96.2	<0.0001	17,049	17.8	499	2.9	16,550	97.1	<0.0001
	Middle school 2nd	15,251	17.4	664	4.4	14,587	95.6		16,743	17.4	595	3.6	16,148	96.4	
	Middle school 3rd	14,906	17.0	726	4.9	14,180	95.1		16,558	17.2	633	3.8	15,925	96.2	
	High school 1st	14,233	16.2	667	4.7	13,566	95.3		15,441	16.1	537	3.5	14,904	96.5	
	High school 2nd	14,617	16.7	639	4.4	13,978	95.6		15,648	16.3	619	4.0	15,029	96.0	
	High school 3rd	14,341	16.4	691	4.8	13,650	95.2		15,558	16.2	622	4.0	14,936	96.0	
Income															
	High	37,724	43.1	1742	4.6	35,982	95.4	<0.0001	35,883	37.4	1263	3.5	34,620	96.5	<0.0001
	Middle	40,058	45.7	1658	4.1	38,400	95.9		48,021	50.0	1639	3.4	46,382	96.6	
	Low	11,498	13.1	592	5.1	10,906	94.9		13,093	13.6	603	4.6	12,490	95.4	
Region															
	Capital area	45,228	51.6	2009	4.4	43,219	95.6	0.4119	49,825	51.9	1790	3.6	48,035	96.4	0.2392
	City area	38,908	44.4	1769	4.5	37,139	95.5		41,253	43.0	1522	3.7	39,731	96.3	
	Rural	5144	5.9	214	4.2	4930	95.8		5919	6.2	193	3.3	5726	96.7	
Smoking															
	Yes	7415	8.5	600	8.1	6815	91.9	<0.0001	3025	3.1	254	8.4	2771	91.6	<0.0001
	No	81,865	93.4	3392	4.1	78,473	95.9		93,972	97.9	3,251	3.5	90,721	96.5	
Alcohol use															
	Yes	13,821	15.8	866	6.3	12,955	93.7	<0.0001	11,832	12.3	672	5.7	11,160	94.3	<0.0001
	No	75,459	86.1	3126	4.1	72,333	95.9		85,165	88.7	2833	3.3	82,332	96.7	
BMI															
	Obese	17,695	20.2	854	4.8	16,841	95.2	0.0016	23,272	24.2	979	4.2	22,293	95.8	<0.0001
	Normal	52,852	60.3	2378	4.5	50,474	95.5		64,531	67.2	2207	3.4	62,324	96.6	
	Underweight	18,733	21.4	760	4.1	17,973	95.9		9194	9.6	319	3.5	8875	96.5	
Physical activity															
	Yes	77,940	89.0	3468	4.4	74,472	95.6	0.4237	68,451	71.3	2414	3.5	66,037	96.5	0.026
	No	11,340	12.9	524	4.6	10,816	95.4		28,546	29.7	1091	3.8	27,455	96.2	
Depressive symptom															
	Yes	17,488	19.6	1121	6.4	16,367	93.6	<0.0001	30,751	31.6	1614	5.2	29,137	94.8	<0.0001
	No	71,792	81.9	2871	4.0	68,921	96.0		66,246	69.0	1891	2.9	64,355	97.1	
Year															
	2017	27,916	31.9	1113	4.0	26,803	96.0	<0.0001	28,851	30.0	890	3.1	27,961	96.9	<0.0001
	2018	27,130	31.0	1089	4.0	26,041	96.0		27,994	29.2	1012	3.6	26,982	96.4	
	2019	15,840	18.1	847	5.3	14,993	94.7		19,150	19.9	794	4.1	18,356	95.9	
	2020	18,394	21.0	943	5.1	17,451	94.9		21,002	21.9	809	3.9	20,193	96.1	

**Table 2:** Results of factors associated with fast food intake.

Variables		Frequent Fast-Food Intake			
		Boys		Girls	
		OR	95% CI	OR	95% CI
Type of household					
	Living with both parents	1.00		1.00	
	Living with single parent	1.08	(0.98–1.19)	1.03	(0.93–1.15)
	Living apart from parents	1.04	(0.80–1.35)	1.18	(0.89–1.62)
	No parents	2.00	(1.52–2.62)	1.58	(1.09–2.30)
Grade					
Middle school	1st	0.89	(0.78–1.01)	0.83	(0.72–0.95)
	2nd	1.00	(0.87–1.14)	0.96	(0.84–1.10)
	3rd	1.10	(0.97–1.25)	1.04	(0.91–1.18)
High school	1st	1.06	(0.94–1.20)	0.92	(0.81–1.05)
	2nd	0.96	(0.85–1.08)	1.07	(0.95–1.22)
	3rd	1.00		1.00	
Income					
	High	1.00		1.00	
	Middle	0.90	(0.90–0.97)	0.96	(0.88–1.05)
	Low	1.01	(1.01–1.13)	1.16	(1.03–1.30)
Region					
	Capital area	1.00		1.00	
	City area	1.03	(0.95–1.11)	0.99	(0.92–1.08)
	Rural	1.00	(0.85–1.19)	0.86	(0.72–1.02)
Smoking					
	Yes	1.69	(1.52–1.89)	1.61	(1.36–1.90)
	No	1.00		1.00	
Alcohol use					
	Yes	1.24	(1.12–1.37)	1.37	(1.22–1.53)
	No	1.00		1.00	
BMI					
	Obese	1.14	(1.05–1.25)	1.32	(1.21–1.44)
	Normal	1.00		1.00	
	Underweight	0.89	(0.81–0.97)	0.99	(0.87–1.13)
Physical activity					
	Yes	1.00		1.00	
	No	1.03	(0.93–1.15)	1.07	(0.98–1.16)
Depressive symptom					
	Yes	1.49	(1.37–1.61)	1.74	(1.61–1.88)
	No	1.00		1.00	
Year					
	2017	1.00		1.00	
	2018	1.04	(0.94–1.15)	1.17	(1.05–1.30)
	2019	1.36	(1.22–1.51)	1.31	(1.17–1.47)
	2020	1.37	(1.23–1.52)	1.26	(1.12–1.41)

**Table 3:** The results of subgroup analysis stratified by independent variables.

Variables	Frequent Fast Food Intake						
	Living with Both Parents	Living with Single Parent		Living Apart from Parents		No-Parents	
	OR	OR	95% CI	OR	95% CI	OR	95% CI
Boys							
Income							
High	1.00	1.22	(1.02–1.46)	1.10	(0.65–1.87)	1.81	(1.16–2.83)
Middle	1.00	1.01	(0.87–1.18)	0.92	(0.61–1.38)	1.64	(0.96–2.78)
Low	1.00	1.04	(0.85–1.27)	1.18	(0.75–1.85)	2.60	(1.67–4.07)
Year							
2017	1.00	1.07	(0.89–1.27)	1.29	(0.81–2.06)	2.35	(1.50–3.70)
2018	1.00	0.93	(0.75–1.15)	0.74	(0.42–1.30)	2.26	(1.47–3.47)
2019	1.00	1.18	(0.95–1.47)	1.19	(0.74–1.92)	1.89	(0.93–3.83)
2020	1.00	1.20	(1.00–1.45)	0.90	(0.51–1.59)	0.71	(0.25–2.04)
Girls							
Income							
High	1.00	1.22	(0.99–1.51)	1.61	(0.94–2.74)	2.30	(1.11–4.80)
Middle	1.00	0.97	(0.83–1.13)	0.99	(0.64–1.52)	0.62	(0.31–1.23)
Low	1.00	0.99	(0.82–1.21)	1.13	(0.69–1.84)	2.13	(1.24–3.64)
Year							
2017	1.00	0.90	(0.72–1.12)	1.07	(0.62–1.84)	1.21	(0.53–2.77)
2018	1.00	0.90	(0.74–1.10)	0.94	(0.56–1.59)	1.93	(0.98–3.82)
2019	1.00	1.23	(0.99–1.51)	1.07	(0.56–2.05)	1.54	(0.85–2.79)
2020	1.00	1.18	(0.95–1.46)	1.90	(1.11–3.25)	1.52	(0.63–3.67)

**Table 4:** Results of factors associated with fast food intake.

Variables	Frequent Fast Food Intake			
	Boys		Girls	
	OR	95% CI	OR	95% CI
Type of household				
Living with both parents	1.00		1.00	
Living with single parent	1.08	(0.98–1.19)	1.03	(0.93–1.15)
Living apart from parents				
Living with family or relative	0.93	(0.66–1.30)	1.23	(0.88–1.71)
Living in dormitory or boarding house	1.15	(0.74–1.78)	1.15	(0.70–1.89)
Living in orphanage	2.07	(0.84–5.12)	0.69	(0.27–1.73)
No parents				
Living with family or relative	1.85	(1.36–2.53)	1.17	(0.73–1.88)
Living in dormitory or boarding house	4.58	(2.27–9.23)	4.40	(1.75–11.08)
Living in orphanage	1.20	(0.49–2.99)	2.17	(0.94–4.99)

## Data Availability

The reports and microdata of KYRBS are released annually in December each year. The KYRBS website [http://yhs.cdc.go.kr, accessed on 1 June 2022] contains microdata files, reports and publications for the survey.
